# A Critical Role of the mTOR/eIF2α Pathway in Hypoxia-Induced Pulmonary Hypertension

**DOI:** 10.1371/journal.pone.0130806

**Published:** 2015-06-29

**Authors:** Ai-ping Wang, Xiao-hui Li, Yong-mei Yang, Wen-qun Li, Wang Zhang, Chang-ping Hu, Zheng Zhang, Yuan-jian Li

**Affiliations:** 1 Department of Pharmacology, School of Pharmaceutical Sciences, Central South University, Changsha, 410078, China; 2 Department of Anatomy, School of Medicine, University of South China, Hengyang, 421001, China; Indiana University, UNITED STATES

## Abstract

Enhanced proliferation of pulmonary arterial vascular smooth muscle cells (PASMCs) is a key pathological component of vascular remodeling in hypoxia-induced pulmonary hypertension (HPH). Mammalian targeting of rapamycin (mTOR) signaling has been shown to play a role in protein translation and participate in the progression of pulmonary hypertension. Eukaryotic translation initiation factor-2α (eIF2α) is a key factor in regulation of cell growth and cell cycle, but its role in mTOR signaling and PASMCs proliferation remains unknown. Pulmonary hypertension (PH) rat model was established by hypoxia. Rapamycin was used to treat rats as an mTOR inhibitor. Proliferation of primarily cultured rat PASMCs was induced by hypoxia, rapamycin and siRNA of mTOR and eIF2α were used in loss-of-function studies. The expression and activation of eIF2α, mTOR and c-myc were analyzed. Results showed that mTOR/eIF2α signaling was significantly activated in pulmonary arteries from hypoxia exposed rats and PASMCs cultured under hypoxia condition. Treatment with mTOR inhibitor for 21 days attenuated vascular remodeling, suppressed mTOR and eIF2α activation, inhibited c-myc expression in HPH rats. In hypoxia-induced PASMCs, rapamycin and knockdown of mTOR and eIF2α by siRNA significantly abolished proliferation and increased c-myc expression. These results suggest a critical role of the mTOR/eIF2αpathway in hypoxic vascular remodeling and PASMCs proliferation of HPH.

## Introduction

Pulmonary hypertension (PH) is a multi-factorial disease with poor prognosis [1]. The disease is progressive and characterized by obstructive remodeling of distal pulmonary arteries, causing an increase in pulmonary vascular resistance and subsequent right ventricular hyperplasia, which ultimately leads to right heart failure and death [2]. Dysregulation in proliferation of pulmonary arterial smooth muscle cells (PASMCs) due to aberrant expression of c-myc and cyclin-dependent kinases (CDKs) are recognized as critical cellular event in pulmonary vascular remodeling [3, 4].

Hypoxia–induced PH (HPH) is one of the most common classes of PH. A number of intracellular signaling mechanisms are observed to be involved in PASMCs proliferation and vascular remodeling in HPH, among which hypoxia-inducible factor 1α (HIF-1α), transforming growth factor (TGF), Wnt and mitogen-activated protein kinases (MAPKs) are widely studied [5–8]. Unfortunately, effective treatment for HPH is still lacking and the identification of new therapeutic targets remains a significant challenge.

Recent research has indicated that the mammalian target of rapamycin (mTOR) in PH is potentially involved in pathogenesis of PAH [9, 10]. Evidence has shown that mTOR contributes to the proliferation and survival of PASMCs in idiopathic pulmonary arterial hypertension (IPAH) in human and experimental PH as well. Chronic hypoxia can lead to mTOR activation [11], while inhibition of mTOR by rapamycin is beneficial for hypoxia- and monocrotaline-induced PH in animal models [11–13]. These results suggest an important role of mTOR in the development of PH. However, the underlying mechanism remains unknown at large.

mTOR is a highly conserved member of the serine/threonine protein kinase family activated by phosphorylation. There is also evidence shows that mTOR plays an important role in regulation of translation initiation [14, 15]. S6K and eIF4E binding protein (4E-BP) are two major substrates of mTOR and are key regulators of protein synthesis by targeting eIF4B, eIF4E, and eukaryotic translation elongation factor 4 (eEF4) [16, 17]. The formation of Met-tRNA and eIF2α complex is a critical step for translation initiation, and eIF2α plays an important role in cell growth and cell cycle regulation through multiple pathways [18–20]. eIF2α is also reported to be important in regulation of airway smooth muscle cell proliferation, hyperplasia and migration [21]. Interestingly, rapamycin is able to prevent eIF2α phosphorylation in hepatic cells [22]. Thus, we hypothesized that eIF2α may play a role in regulation of PASMCs proliferation, and activation of eIF2α might be modulated by mTOR.

In summary, the purpose of this study was to explore the contribution of mTOR and eIF2α in vascular remodeling in HPH rat model, and the role of the mTOR/eIF2α pathway in hypoxia induced PASMCs proliferation and vascular remodeling in PH.

## Materials and Methods

### Animals

Male Sprague–Dawley rats (weighing 180–220 g) were obtained from the Laboratory Animal Center, Xiangya School of Medicine, Central South University (Changsha, China). All surviving animals were handled in accordance with the National Institutes of Health Guide for the Care and Use of Laboratory Animals. The experimental protocol was approved by the medicine animal welfare committee of Xiangya Medical School, Central South University (Changsha, China).

### Animal experiments

Rats were randomly divided into three groups (n = 10/group): (i) normoxia group; (ii) hypoxia group, and (iii) hypoxia plus rapamycin group. Rats in the hypoxia or hypoxia plus rapamycin groups were placed in a chamber and exposed to 10% O2 continuously for 21 days, and rats in the hypoxia plus rapamycin group were administrated with rapamycin (1 mg/kg/d, oral gavage) from the first day of their exposure to hypoxia for 21 days. On day 22, animals were anesthetized with sodium pentobarbital (30 mg/kg, i.p.), right ventricle systolic pressure (RVSP) and mean pulmonary artery pressure (mPAP) were monitored. After sacrificing the animals, the right and left ventricle (RV, LV) and the interventricular septum (S) were dissected. The tibia length was measured and weighed for calculating the ratio of RV to (LV+S) and tibia length/RV, which are the key indexes used for evaluating RV hyperplasia. The pulmonary arterial samples were isolated for determination of mRNA expression. Portions of excised lungs were fixed in 4% paraformaldehyde for hematoxylin–eosin and immunohistochemical staining. The remainder of the excised lungs was frozen in liquid nitrogen for immunofluorescent staining.

### Histology, immunohistochemistry and immunofluorescent

Vascular remodeling of lung tissues was studied. Lung hematoxylin–eosin staining was performed as previously described [23]. Slices of arteries at a thickness of 3 μm were stained with hematoxylin-eosin staining. Pulmonary arterial wall thickness (WT) was calculated by the following formula: WT (%) = area_ext_−area_int_/ area_ext_×100, where area_ext_ and area_int_ are the area bounded by external and internal elastic lamina, respectively. For immunohistochemistry and immunofluorescent staining, sections were stained with anti-eIF2α antibody (ab131495, 1:100; abcam), anti-p-eIF2α antibody (#3398, 1:100; Cell Signaling Technology), anti-mTOR antibody (#2972, 1:100; Cell Signaling Technology), anti-p-mTOR antibody (#2974, 1:100; Cell Signaling Technology), anti-c-myc antibody (#5605, 1:100; Cell Signaling Technology), anti-α-SMA(ab7817; 1:200; abcam), developed with DAB, and counterstained with hematoxylin. The immunohistochemistr**y** samples were treated with horseradish peroxidase (HRP)-conjugated goat anti-rabbit secondary antibodies (ZB-2301, 1:500) for 1 h at 37°C. Images were acquired using a Nikon Olympus BX51. The immunofluorescence samples were treated with cy3-conjugated (BA1032, 1:50) and/or fluorescein isothiocyanate (FITC)-conjugated secondary antibodies (BA1101, 1:50) for 1 h at 37°C. Analysis of the intensity in smooth muscle actin (SMA)-positive areas of small muscular PAs was performed using an Olympus BX51.

### Cell cultures

PASMCs were isolated from the pulmonary artery of 10-week-old male Sprague-Dawley rats using the method described previously [24]. The cells were cultured at 37°C under 5% CO_2_ in Dulbecco’s modified Eagle’s medium (DMEM) containing 20% fetal bovine serum (FBS). PASMCs were identified by immunohistochemical staining using an antibody against smooth muscle α-actin (ab7817, 1:50; Abcam, Hong Kong, China). PASMCs between passages 3 and 8 were used for the experiments.

Three series of experiments were designed. The first series of experiments investigated the role of hypoxia on PASMCs proliferation. PASMCs were subjected to hypoxia for 12, 24, 48, 60 and 72 h to determine the optimal duration of exposure to hypoxia. The second series of experiments were designed to explore the effect of eIF2α siRNA on PASMCs proliferation and the involvement of c-myc pathway. PASMCs were treated with eIF2α siRNA (10, 25, 50 nmol/L) under normal culture conditions for 4 h, then subjected to hypoxia (3%O_2_, 5%CO_2_ and 92%N_2_) for 48 h. Cell proliferation assays were performed. eIF2α and c-myc expression were analyzed. The 48 h hypoxia duration was determined based on previous pilot study. The three series of experiments were designed to explore the effect of rapamycin (RAPA) on PASMCs proliferation and involvement of the eIF2α/c-myc pathway. PASMCs were treated with rapamycin (10, 20, 40 and 80 nmol/L), then subjected to hypoxia (3%O_2_, 5%CO_2_ and 92%N_2_) for 48 h. Cell proliferation assays were performed and the expression of eIF2α, mTOR and c-myc was analyzed.

### Small interference RNA transfection

The mTOR and eIF2α siRNA were purchased from Ribobio (Guangzhou, China). TurboFect transfection reagent (#0531; Thermo scientific, USA) were used for cell transfection. PASMCs grown to 60% to 70% confluence were starved with 2% fetal bovine serum in Dulbecco’s modified Eagle’s medium (DMEM) for 24 h and then transfected with Turbofect transfection reagent for 4–6 h, and then fresh medium were replaced according to the manufacturer’s instruction. Cells were harvested 4h after for the experiments as mentioned above. Transfection efficiency was evaluated by eIF2α mRNA and protein expression using real-time PCR and Western blot analysis, respectively. Each experiment was repeated using primarily cultured cells (3–8 passage). The experiments were repeated three times.

### DNA synthesis analysis

DNA synthesis analysis was performed using CellTiter 96 AQ_ueous_ One Solution cell Proliferation Assay (MTS; Promega, USA) according to the manufacturer’s instruction. CellTiter 96 AQueousOne Solution Cell Proliferation Assay is a colorimetric method for determination of cell viability in proliferation or cytotoxicity assays. PASMCs were plated at a dense of 6,000 cells/well in 96-well plates. After the cells were attached to the 96-well plates, the medium was replaced with DMEM culture supplemented with 2% fetal bovine serum and incubated for 24 h. The first experiment was designed to determine the effect of hypoxia on the proliferation of PASMCs. Cells were subjected to hypoxia for 12, 24, 48, 60, or 72 h, cultured, and then incubated for 2 h with 20 μl/well MTS. Absorbance was measured at 490 nm (DTX880; Beckman, Miami, FL). To determine the effect of eIF2α siRNA on proliferation of PASMCs, cells were exposed to hypoxia for 48 h in the presence or absence of eIF2α siRNA (10, 25, 50, 100 nmol/l) for 4 h, 20 μl/well MTS was added followed by incubation for 2 h. Absorbance was measured at 490 nm. All experiments were performed in triplicate and repeated at least three times.

### RNA preparation and quantitative reverse transcription PCR analysis

Total RNA was extracted by TRIzol (Invitrogen, Carlsbad, CA). For detection of mRNAs and miRNAs expression, RNA (0.2–0.5 μg) was subjected to reverse transcription reaction using the PrimeScript reverse transcription reagent Kit (DRR037A; TaKaRa, Dalian, China) according to the manufacturer’s instructions. Quantitative analysis of mRNAs and miRNA expression was performed using SYBR Premix Ex Taq (DRR420A; TaKaRa) at ABI 7300 system. PCR cycling conditions were as follows: an initial incubation at 95°C for 15 s, followed by 40 cycles of denaturation at 95°C for 5 s, and annealing at 60°C for 31 s. For detection of mature miRNAs, Bulge-Loop miRNA Primers (Ribobio) were used. The sequences of oligonucleotides used were as follows: Primers for eIF2α were: 5′- GGACAAATGGAAGTATGGGATG-3′ (forward), 5′-CAAGAGAGAGCCAGTGTAATGC-3′ (reverse). Primers for mTOR were: 5′- ATCCAGACCCTGACCCAAAC-3′(forward), 5′- TCCACCCACTTCCTCATCTC -3′(reverse). Primers for c-myc were: 5′- TGTCCGTTCAAGCAGATGAG -3′(forward), 5′- CGGTCAGTTTATGCACCAGA -3′(reverse). Primers for β-actin were: 5′-TGTCACC-AACTGGGACGATA-3′(forward), 5′-ACCCTCATAGATGGGCACAG-3′(reverse). Data analysis was performed by comparative Ct method using the ABI software. U6 and β-actin were used to normalize the expression of mRNA and miRNAs, respectively. The results were repeated at least three times independently using three different pools of templates.

### Western blot analysis

Protein was extracted from PASMCs with RIPA buffer (contain 0.1% PMSF), and equal amounts of protein from each sample (20 or 40 μg) were separated by 12% sodium dodecyl sulfate polyacrylamide gel electrophoresis and transferred to polyvinylidine fluoride membranes. The membranes were then incubated with primary antibodies overnight at 4°C and horseradish peroxidase-coupled goat anti-rat secondary antibody (ZB-2301, 1:5,000; Beijing, China). The chemiluminescence signals were detected with the EasySee Western Blot Kit (Beijing TransGen Biotech, Beijing, China). The densitometric analysis was conducted with Image J 1.43 (National Institutes of Health). Primary antibodies against eIF2α (ab131495, 1:500) were purchased from abcam (Hong Kong, China). Primary antibodies against p-eIF2α (#3398, 1:1000), c-myc (#5605, 1:1000), mTOR (#2972, 1:1000), and p-mTOR (#2974, 1:1000) were obtained from Cell Signaling Technology. Primary antibody against β-actin (BM0627, 1:250) was purchased from Boster.

### Data analysis

Data were presented as the mean ± S.E.M (standard errors). Statistical analysis was performed by unpaired Student’s *t*-test for two groups or analysis of variance followed by Newman–Student–Keuls test for multiple groups. Statistical significance was defined as *P*<0.05.

## Results

### mTOR was activated in hypoxia-induced PH rats and primary PASMCs

**Fig 1:**
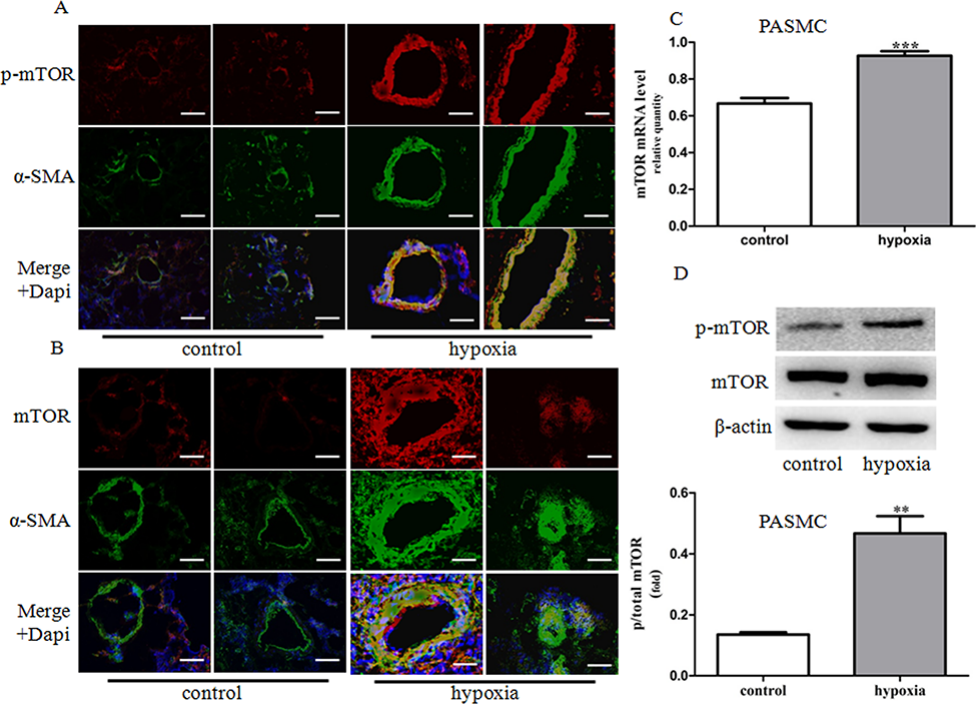
Expression and activity of mTOR in pulmonary arteries from HPH rats and PASMCs under hypoxia. (A-B) Dual immunofluorescence analysis with anti-p-mTOR (red), anti-mTOR (red) and anti-SMA antibodies (green) in lung tissue specimens in rats. Images were acquired with an Olympus BX51 microscope, scale bars = 100um. (C) The mRNA expression of mTOR was determined by real-time PCR in primary PASMCs. (D) The expression and phosphorylation of mTOR proteins was determined by western blot in PASMCs. Control: cells were treated with 21% O_2_ for 48 h. Hypoxia: cells were treated with 3% O_2_ for 48 h. Data represent fold changes in p-mTOR/β-actin protein ratios. Data are means ±S.E.M. **P<0.01 vs. control; ***P<0.001 vs. control.

Immunofluorescence analysis of lung tissues from hypoxia-induced PH rats showed a significant increase in p-S2481-mTOR expression level, a marker for mTOR catalytic activity. The expression of mTOR was located in SMA-positive areas in small remodeled pulmonary arteries in lungs of hypoxia-induced PH rats, suggesting a role of mTOR in medial hyperplasia of pulmonary arteries (Fig 1A and 1B). PASMCs cultured under hypoxia condition demonstrated a significant increase in phosphorylation of mTOR and mTOR mRNA expression (Fig 1C and 1D). These data indicated that hypoxia can induce mTOR activation in primarily cultured PASMCs, which may contribute to pulmonary vascular medial hyperplasia.

### Activation of eIF2α in hypoxia-induced PH rats and primarily cultured PASMCs accompanied by up-regulation of c-myc expression

**Fig 2:**
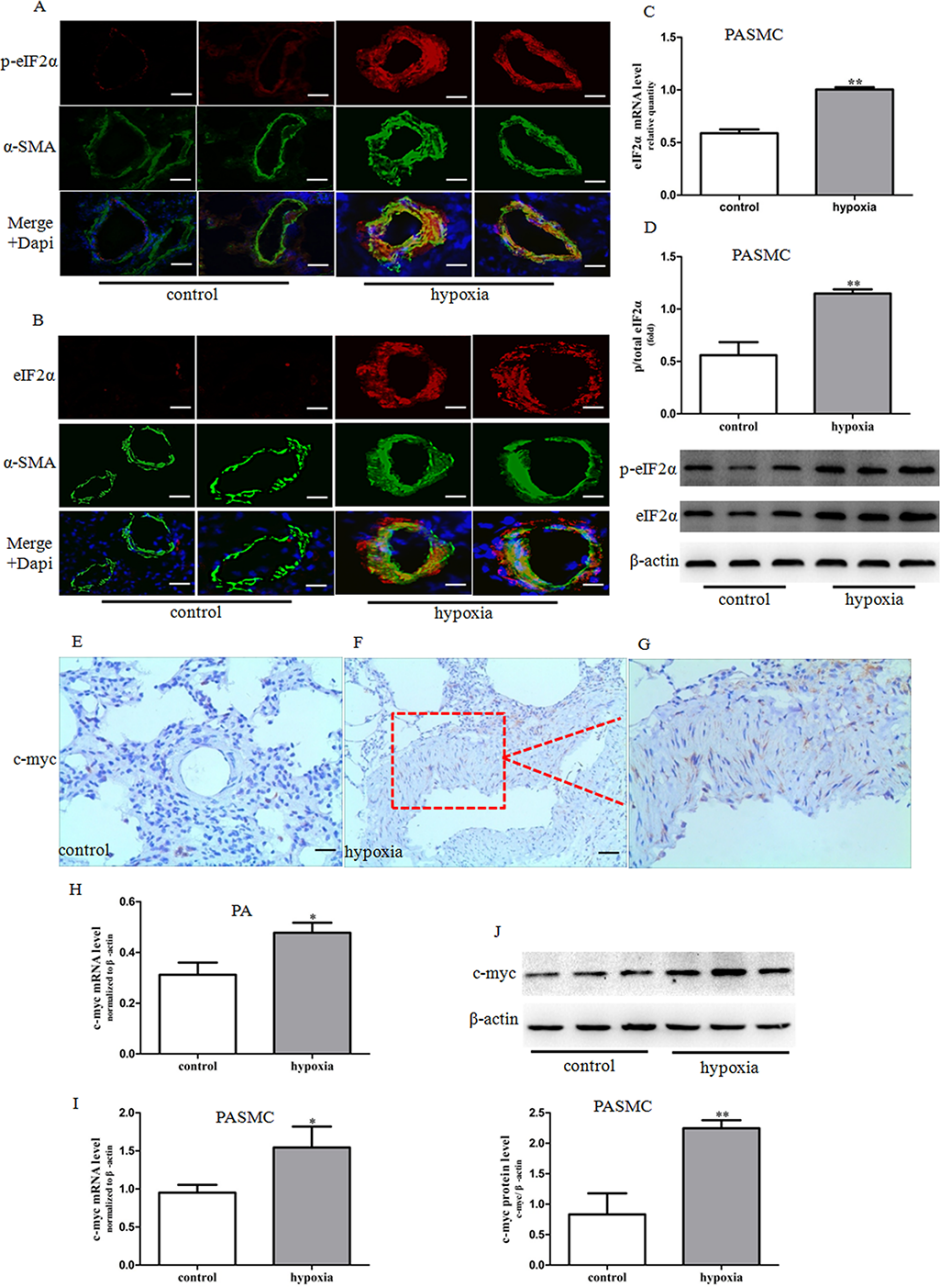
Expression and activity of eIF2α and c-myc expression in pulmonary arteries from HPH rats and PASMCs under hypoxic conditions. (A-B) Dual immunofluorescence analysis with anti-p-eIF2α (red), anti-eIF2α (red) and anti-SMA antibodies (green) in lung tissue specimens in rats. Images were acquired with an Olympus BX51 microscope, scale bars = 100um. (C) The mRNA expression of eIF2α was determined by real-time PCR in PASMCs. (D) The phosphorylation of eIF2α proteins was determined by western blot in PASMCs. Data represent fold changes in p-eIF2α/total eIF2αprotein ratios. (E-G) The expression of c-myc protein was determined by immunohistochemistry staining (brown precipitate indicates c-myc positive staining). Images (E, F) were taken under ×20 magnification. G is an enlarged portion of the red box in E. (H) The expression of c-myc mRNA was determined by real-time PCR in pulmonary arteries. (I) The expression of c-myc mRNA was determined by real-time PCR in PASMCs. (J) The expression of c-myc protein was determined by western blot in PASMCs. Control: cells were treated with 21% O_2_ for 48 h. Hypoxia: cells were treated with 3% O_2_ for 48 h. Data are means ±S.E.M. n = 3. *P<0.05 vs. control; **P<0.01 vs. control.

eIF2α is a critical protein for cell growth and cell cycle regulation. A recent study demonstrated that eIF2α is involved in the regulation of airway smooth muscle cell proliferation, hyperplasia and migration [21]. Thus, we hypothesized that eIF2α may be involved in the regulation of PASMCs proliferation. Immunofluoresence analysis of lung tissue from hypoxia-induced PH rats showed a marked increase in eIF2α phosphorylation. The expression of eIF2α was located in SMA-positive areas in small remodeled pulmonary arteries in hypoxia-induced PH lungs (Fig 2A and 2B) as observed for mTOR, suggesting a possible link between eIF2α and PASMCs proliferation and medial hyperplasia. And also, primarily cultured PASMCs from SD rats demonstrated a significant elevation of p-eIF2α under hypoxic conditions, which persisted for up to 48 h (Fig 2C and 2D).

c-myc is an important member of the myc family and is involved in the regulation of cell proliferation and differentiation [25]. Our previous study had shown that c-myc is involved in hypoxia-induced PASMCs proliferation [24]. Results from both immunohistochemical analysis and western blot showed elevated c-myc expression in pulmonary arteries from HPH rats and in PASMCs cultured under hypoxia condition (Fig 2E–2H, Fig 2I and 2J).

### mTOR inhibition attenuated vascular remodeling in HPH rats and inhibited hypoxia-induced PASMCs proliferation

**Fig 3:**
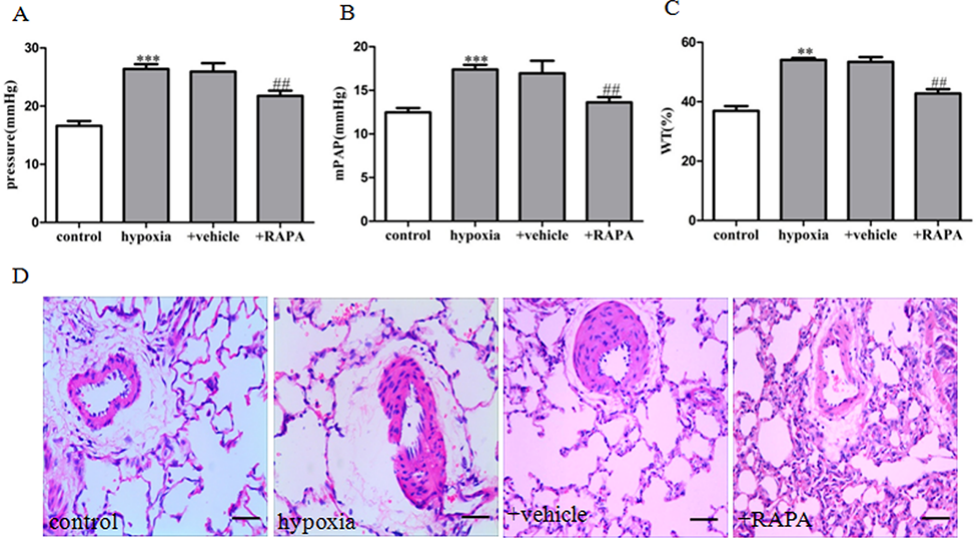
Effect of rapamycin on hemodynamic changes and vascular remodeling in HPH rats. (A) Right ventricle systolic pressure (RVSP). (B) Mean pulmonary artery pressure (mPAP). (C) Statistical graph analysis of pulmonary arterial media wall thickness (WT). (D) Hematoxylin-eosin staining in small PAs. Data are means ± S.E.M. n = 8. RAPA: rapamycin. **P<0.01 vs. control; ***P<0.001 vs. control; ^##^ *P*<0.05 vs. hypoxia.

In keeping with a previous study [26], three weeks of exposure to hypoxia induced pulmonary hypertension in rats, as demonstrated by a significant elevation in RVSP and mPAP compared with the control rats (Fig 3A and 3B). Hypoxia also significantly induced hyperplasia of pulmonary arteries, compared with controls (Fig 3C and 3D). The elevation in RVSP and mPAP were decreased by mTOR inhibitor rapamycin (Fig 3A and 3B). Rapamycin treatment also attenuated hyperplasia in the vascular media of small pulmonary arteries (Fig 3C and 3D). Treatment with rapamycin also reversed hypoxia-induced PASMCs proliferation (Fig 5G and 5H). Taken together, these results showed that the mTOR inhibitor rapamycin attenuates vascular remodeling of HPH, most likely through inhibition of PASMCs proliferation.

### mTOR inhibition prevents eIF2α expression and activity

**Fig 4:**
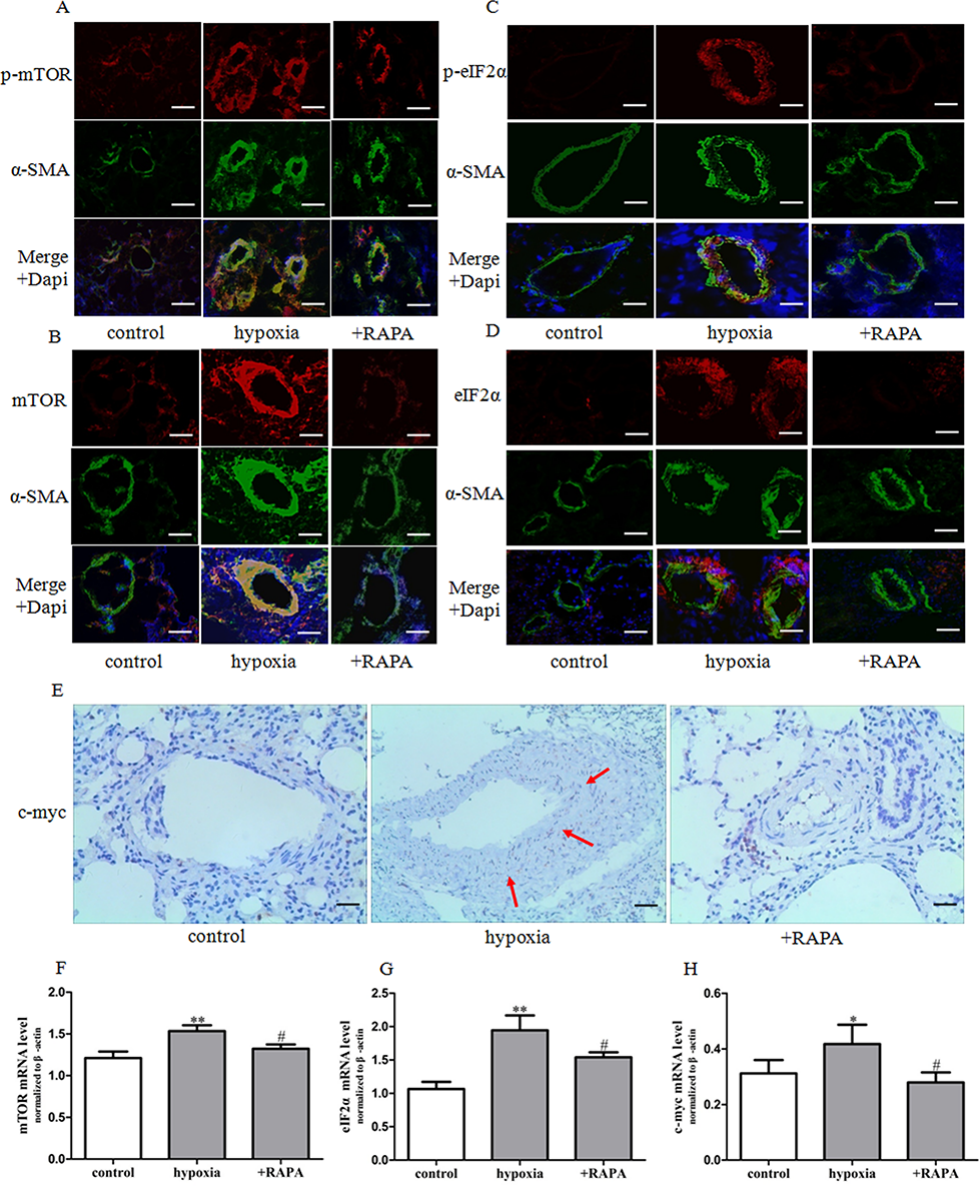
Effect of rapamycin on mTOR, eIF2α and c-myc expression and activity in HPH rats. (A-D) Dual immunofluorescence analysis with anti-p-mTOR (red), anti-mTOR (red), anti-p-eIF2α (red), anti-eIF2α (red), and anti-SMA antibodies (green) in lung tissue specimens in rats. Images were acquired with an Olympus BX51 microscope, scale bars = 100um. (E) The expression of c-myc protein was determined by immunohistochemistry staining (brown precipitate indicates c-myc positive staining) scale bars = 200um. (F-H) The expression of mTOR, eIF2α and c-myc mRNA was determined by real-time PCR in pulmonary arteries. Data are means ± S.E.M. n = 8. RAPA: rapamycin. *P<0.05 vs. control; **P<0.01 vs. control; ^#^ *P*<0.05 vs. hypoxia.

**Fig 5:**
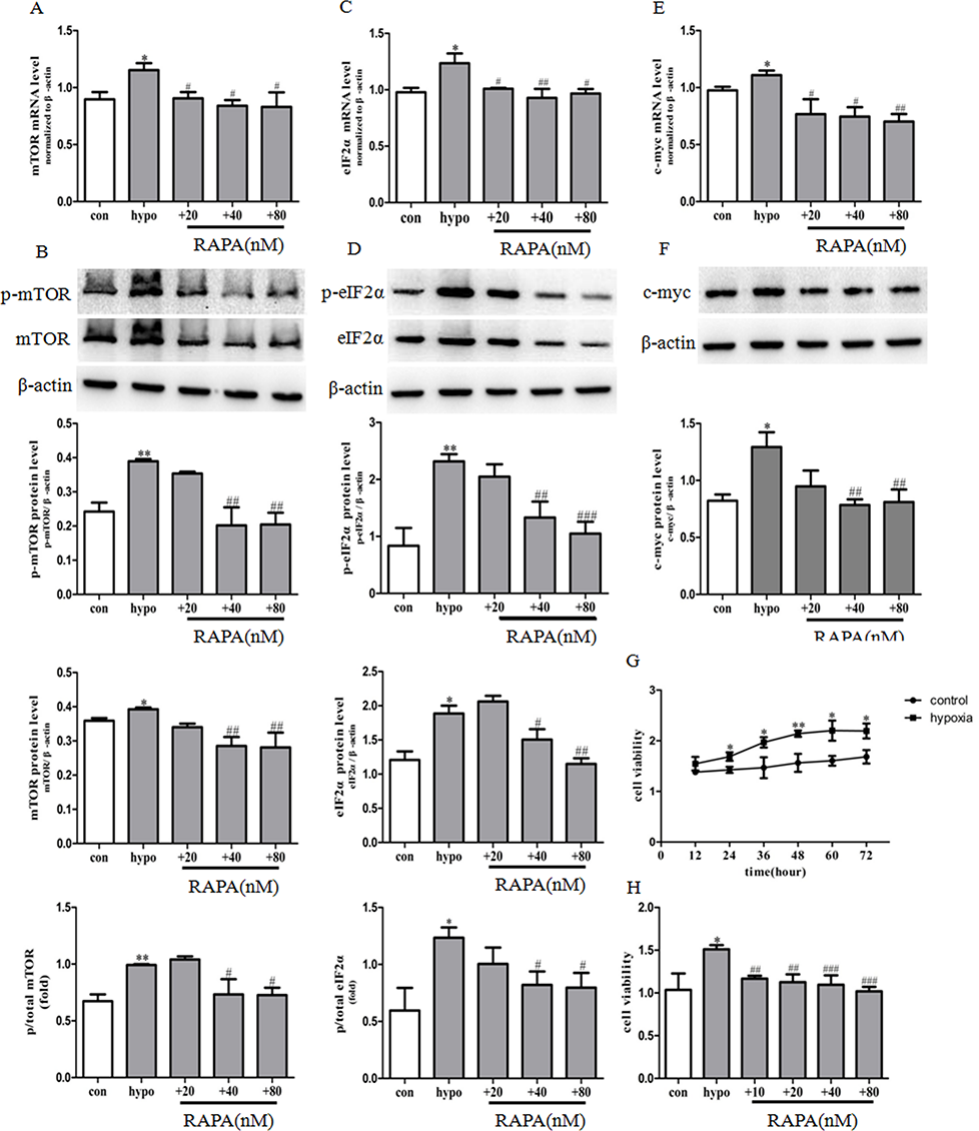
Rapamycin prevented mTOR, eIF2α and c-myc expression and PASMCs proliferation induced by hypoxia. The mRNA expression of mTOR (A), eIF2α (C) and c-myc (E) was determined by real-time PCR. The protein expression of p-mTOR and mTOR (B), p-eIF2α and eIF2α (D), c-myc (F) was determined by western blot. (G) Proliferation of PASMCs was measured by MTS assay after incubation with hypoxia for 12, 24, 36, 48, 60, 72h. (H) Cell proliferation was measured by MTS assay. control: cells were treated with 21% O_2_ for 48 h. hypoxia: cells were treated with 3% O_2_ for 48 h; +RAPA 10–80 nM: cells were treated with RAPA 10–80 nM and then subjected to 3% O_2_ for 48 h. Data are means ± S.E.M. n = 3. RAPA: rapamycin. *P<0.05 vs. con; **P<0.01 vs. con; ^#^ *P*<0.05 vs. hypo; ^##^ *P*<0.01 vs. hypo; ^###^ *P*<0.001 vs. hypo.

**Fig 6:**
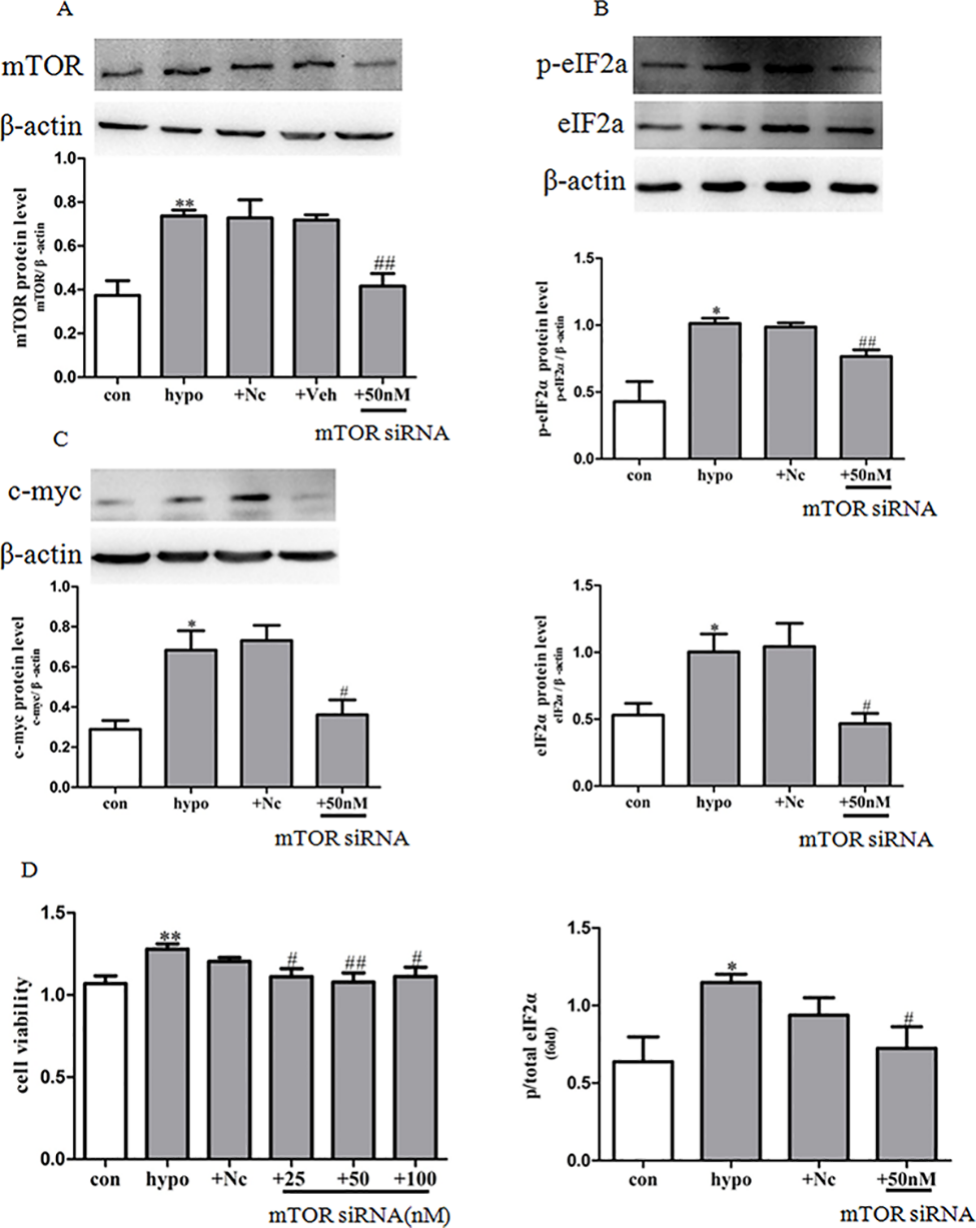
mTOR siRNA inhibited mTOR, eIF2α and c-myc expression and PASMCs proliferation induced by hypoxia. The protein expression of mTOR (A), p-eIF2α and eIF2α (B), c-myc (C) was determined by western blot, statistical result of total gray value was estimated by the image analysis software. (D) Proliferation of PASMCs was measured by MTS assay. con: cells were treated with 21% O_2_ for 48 h; hypo: cells were treated with 3% O_2_ for 48 h; +Nc: cells were transfected with negative control 50nM for 4 h before stimulation with hypoxia for 48 h; +Veh: cells were transfected with transfection reagent (30uL/6 well) for 4 h before stimulation with hypoxia for 48 h; +mTOR siRNA 50 nM: cells were transfected with mTOR siRNA 50 nM for 4 h before stimulation with hypoxia for 48 h. Data represent the means ±S.E.M. n = 3. *P<0.05 vs. con; **P<0.01 vs. con; ^#^ *P*<0.05 vs. hypo; ^##^ *P*<0.01 vs. hypo.

As observed in our study, mTOR and eIF2α were up-regulated both in the media of pulmonary arteries and in hypoxia-induced cultured PASMCs. We next determined whether mTOR contributes to PASMCs proliferation and pulmonary vascular remolding via eIF2α. We performed immunofluorescence staining and immunohistochemical analysis of lung tissues from rats with chronic hypoxia-induced pulmonary vascular remodeling. Rapamycin markedly decreased mTOR, p-eIF2α and eIF2α levels (Fig 4A, 4D, 4F and 4G). At the same time, rapamycin decreased p-mTOR, p-eIF2α and eIF2α levels, and reduced cell proliferation in PASMCs under hypoxia (Fig 5A and 5B, 5D and 5E, 5G). In addition, p-eIF2α and total eIF2x expression was decreased and PASMCs proliferation by hypoxia-induced was inhibited after mTOR knockdown by siRNA (Fig 6A and 6B, 6D and 6E). These results were consistent with those obtained from the use of the mTOR pharmacologic blocker rapamycin, which strongly suggested that mTOR may act as an upstream positive regulator of eIF2α.

### c-myc expression and proliferation of PASMCs is regulated by mTOR and eIF2α

**Fig 7:**
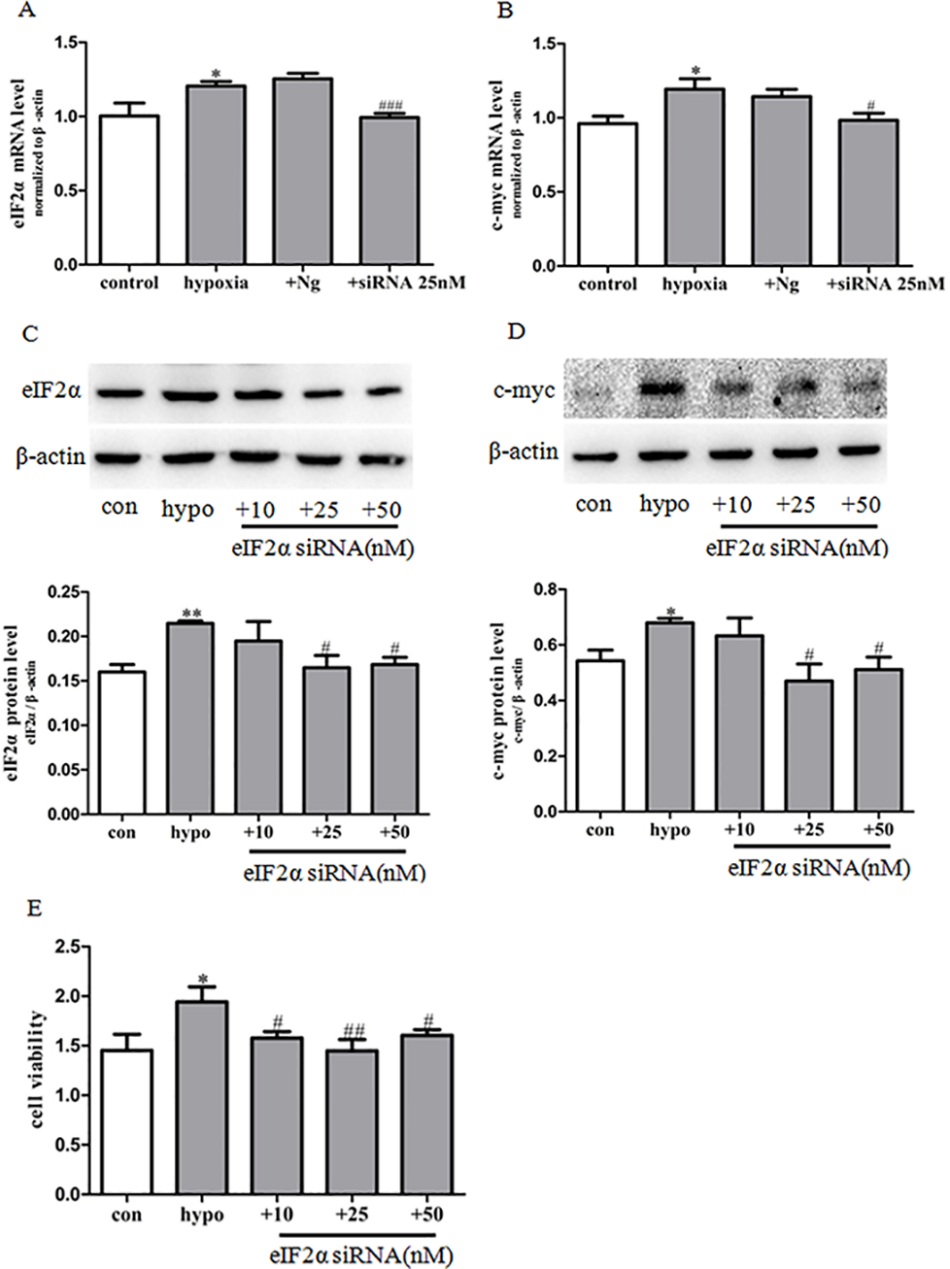
Effect of eIF2α siRNA on c-myc expression and PASMCs proliferation induced by hypoxia. (A) The expression of eIF2α mRNA was determined by real-time PCR. (B) The expression of c-myc mRNA was determined by real-time PCR. Data represent the means ±S.E.M. n = 3. *P<0.05 vs. control; ^#^ *P*<0.05 vs. hypoxia; ^###^ *P*<0.001 vs. hypoxia. (C) The expression of eIF2α protein was determined by western blot. Statistical result of total gray value was estimated by the image analysis software. (D)The expression of c-myc protein was determined by western blot. Statistical result of total gray value was estimated by the image analysis software. PASMCs were transfected with the siRNA of eIF2α or negative control (Nc) 4 h before stimulation with hypoxia for 48 h. (E) Proliferation of PASMCs was measured by MTS assay. con: cells were treated with 21% O_2_ for 48 h. hypo: cells were treated with 3% O_2_ for 48 h; +eIF2α siRNA 10–50 nM: cells were transfected with eIF2α siRNA 10–50 nM for 4 h before stimulation with hypoxia for 48 h. Data represent the means ±S.E.M. n = 3. *P<0.05 vs. con; ^#^ *P*<0.05 vs. hypo; ^##^ *P*<0.01 vs. hypo; ^###^ *P*<0.001 vs. hypo.

We next determined whether mTOR and eIF2α can control c-myc expression and cell proliferation. Our results showed that rapamycin markedly decreased c-myc expression *in vivo* and *in vitro* (Figs 4E, 4H, 5C and 5F), and mTOR knockdown by siRNA obviously decreased c-myc expression in cultured PASMCs (Fig 6C). Furthermore knockdown of eIF2α by siRNA was conducted in PASMCs. Firstly, we confirmed the efficiency of siRNA knockdown by western blot (Fig 7A and 7C). Secondly, we found that eIF2α siRNA treatment suppressed hypoxia-induced increase in c-myc expression and PASMCs proliferation markedly (Fig 7B, 7D and 7E). Taken together, these data demonstrated that activation of eIF2α and upregulation in c-myc expression are critical downstream events to mTOR that contribute to PASMCs proliferation and vascular remodeling in HPH.

## Discussion

Dysregulation in proliferation and migration of PASMCs in small pulmonary arteries are critical components of pulmonary vascular remodeling and lead to medial hyperplasia in PH [27, 28, 9, 11]. Activation in mTOR signaling has been recognized as a hallmark of vascular remodeling in PH. Our present work further confirms the beneficial effect of mTOR/eIF2α inhibition by rapamycin in HPH rats and provides a novel mechanistic link between mTOR/eIF2α pathway and PASMCs proliferation and vascular remodeling in HPH.

Vasoconstriction and vascular remodeling are two major pathological characteristics of PH. Current medicines for the treatment of PH such as nitric oxide (NO), endothelin 1 receptor (ET1R) antagonist, prostacyclin and sildenafil are focused on improving vasodilatation. However, preventing vascular remodeling is supposed to have a greater impact on improving prognosis [1]. Several intracellular signaling proteins including mammalian target of rapamycin (mTOR), hypoxia- inducible factor 1α (HIF-1α), transforming growth factor (TGF) have been linked to PASMCs proliferation and vascular remodeling in PH [29, 13, 5, 7]. Research on the efficiency and safety of treatments targeting these signaling pathways has already been carried out in experimental PH models.

A critical role of mTOR signaling in cellular function has been reported mainly in cancer cells [30]. Emerging evidence has shown that mTOR is also involved in the regulation of PASMCs proliferation and survival in human IPAH and experimental PH [31, 11]. Increased expression and activity of mTOR has been observed in pulmonary arteries from monocrotaline and hypoxia-induced PH models [32–34]. Our results showed that three weeks exposure to hypoxia up-regulated mTOR expression and phosphorylation in pulmonary vessels. Further, immunofluorescence histochemistry showed co-expression of mTOR and α-SMA, a marker of smooth muscle cells. *In vitro* experiments showed that chronic hypoxia induced mTOR activation in primarily cultured rat PASMCs. These results suggest that mTOR is activated in PASMCs proliferation and pulmonary vascular remodeling in HPH. To further investigate whether activated mTOR is involved in the progression of PH, the mTOR inhibitor rapamycin was used *in vivo* and *in vitro*. Our findings showed that rats treated with rapamycin were resistant to chronic hypoxia, as evidenced by lower right ventricle systolic pressure (RVSP) and mean pulmonary arterial pressure (mPAP) and thinner pulmonary vessel walls. Pretreatment with rapamacin or knockdown of mTOR by siRNA also abolished hypoxia-induced proliferation of PASMCs. Collectively, our results demonstrate that mTOR plays an important role in PASMCs proliferation and vascular remodeling in HPH.

Results from our lab and other scientists [35–38] provide conclusive evidence that mTOR inhibition is beneficial for PH and that mTOR signaling could represent a new therapeutic target for PH treatment. However the exact underlying mechanism is still not very clear. Two forms of the mTOR complex, mTORC1 (mTOR-raptor) and mTORC2 (mTOR-rictor), are identified. mTORC1 is sensitive to rapamycin and is thought to mainly function in cell growth and the regulation of proliferation, while mTORC2 usually play a role in cell structure and can also be affected by long-term rapamycin treatment [31, 39]. Recently, Goncharov DA [11] et al found that mTORC2 plays a coordinating role in the metabolism, proliferation and survival of PASMCs in PAH. Krymskaya VP et al have shown that both mTORC1 and mTORC2 are required for PASMCs proliferation induced by chronic hypoxia in vitro and in vivo. Previous studies have revealed that S6K and eIF4E binding protein (4E-BP1) are major downstream effectors of mTOR. But more details on mechanism of mTOR activation requires further investigation.

The present study suggested that eIF2α acts as a new mediator of mTOR and an important regulator of PASMCs proliferation in HPH. Firstly, under hypoxic conditions, mTOR activation was accompanied by up-regulation of eIF2α expression and activity (as determined by phosphorylation) in pulmonary arteries and PASMCs. Secondly, rapamycin treatment significantly reversed the activation of eIF2α in vitro and in vivo. Thirdly, knockdown of mTOR with siRNA reversed the increase in eIF2α expression and phosphorylation in hypoxia-induced PASMCs. In addition to eIF2α, other translation initiation factors are also involved in mTOR signaling. For example, mTOR can bind to eIF3 and lead to release and activation of S6K. 4E-BP1 is one of the main downstream proteins of mTOR [40, 17]. All these results confirmed a critical role of mTOR signaling in protein translation process. Notably, although the present results give a clue that the effects of mTOR inhibitor might be mediated at least in part by eIF2α down-regulation, studies on the causal relationship of eIF2α and mTOR is still lacking. The mechanism by which mTOR was activated and which complex of mTOR activates eIF2α in PASMCs should be fully revealed in future study.

Anyway, a critical role of eIF2α in PASMCs proliferation and vascular remodeling in HPH enlarged our understanding on the role of eIFs in PH. In this study, we observed a marked increase in p-eIF2α, a marker for eIF2α catalytic activity, in SMA-positive areas in small muscular remodeled vessels of lungs of HPH rats. Importantly, we found that eIF2α siRNA prevented hypoxia-induced PASMCs proliferation. It suggested that eIF2α might be crucial for the media hyperplasia in HPH rat through stimulation of PASMCs proliferation. We think it is only the first step to reveal the role of eIFs in PH, investigating the effect of other eIFs including eIF3, eIF4 on PASMCs proliferation and vascular remodeling would be helpful to decipher the role of eIFs in PH and provide more evidence for potential therapeutical strategy targeting eIFs.

In this study, we found that eIF2α siRNA can reverse hypoxia-induced PASMCs proliferation and c-myc expression. However, much work remains to be done to elucidate the mechanism underlying eIF2α regulation of PASMCs proliferation. Previous studies showed that activation of c-myc is considered to be one of the initiating steps in vascular smooth muscle cell proliferation. An aberrant expression of c-myc is observed in injured vessels after angioplasty [41]. Consistent with previous research, c-myc expression was up-regulated in pulmonary arteries and PASMCs exposed to hypoxia and was inhibited by either mTOR or eIF2α inhibition. Proteins that regulate cell proliferation and differentiation such as c-myc may be important downstream targets of eIF2α in HPH. However, further studies with eIF2α knockdown in vivo and c-myc siRNA treatment or overexpression would help decipher the role of eIF2α in PH.

## Conclusions

Our findings suggest a critical role of the mTOR/eIF2α pathway in hypoxic vascular remodeling and PASMCs proliferation in HPH. Inhibition of mTOR/eIF2α pathway could serve as a new solution for the treatment of PH.

## Supporting Information

S1 Fig Cellular proliferation stimulated by 20% FBS. Cellular proliferation stimulated by 20% FBS (48 hours) was assayed in PASMCs treated with siRNA against mTOR or eIF2α, or in the presence of rapamycin. As shown in MTS experiments, cell proliferation was inhibited by either rapamycin (10, 20, 40, 80 nmol/L) (A), eIF2α siRNA (25, 50, 100 nmol/L) (B), or mTOR siRNA (25, 50, 100 nmol/L) (C). These results are in agreement with cell proliferation data acquired with hypoxia. Data represent the means ±S.E.M. n = 3. *P<0.05 vs. con; **P<0.01 vs. con; ***P<0.001 vs. con; ^#^ *P*<0.05 vs. hypoxia; ^##^ *P*<0.01 vs. hypoxia. The above experiments were repeated three times with similar results.
(TIF)

S2 Fig mRNA expression of Ki-67 and PCNA in pulmonary arteries. (A) The expression of Ki-67 mRNA was determined by real-time PCR. (B) The expression of PCNA mRNA was determined by real-time PCR. Data represent the means ±S.E.M. n = 3. *P<0.05 vs. control; **P<0.01 vs. control; ^#^ *P*<0.05 vs. hypoxia; ^##^ *P*<0.01 vs. hypoxia. The above experiments were repeated three times with similar results.
(TIF)

S3 Fig Schematic representation of proposed function of mTOR/eIF2α pathway in PASMCs in HPH. (TIF)

## References

[1] Morrell NW, Adnot S, Archer SL, Dupuis J, Jones PL, Maclean MR, et al (2009) Cellular and Molecular Basis of Pulmonary Arterial Hypertension. J Am Coll Cardiol 54(1 Suppl):S20–31. 10.1016/j.jacc.2009.04.018 19555855 PMC2790324

[2] Ricard N, Tu L, Le Hiress M, Huertas A, Phan C, Thuillet R, et al (2014) Increased Pericyte Coverage Mediated by Endothelial-Derived Fibroblast Growth Factor-2 and Interleukin-6 Is a Source of Smooth Muscle–Like Cells in Pulmonary Hypertension. Circulation 129(15): 1586–97. 10.1161/CIRCULATIONAHA.113.007469 24481949

[3] Sakao S, Tatsumi K. (2011) Vascular remodeling in pulmonary arterial hypertension: multiple cancer-like pathways and possible treatment modalities. Int J Cardiol 147(1): 4–12. 10.1016/j.ijcard.2010.07.003 20692712

[4] Masri FA, Xu W, Comhair SAA, Asosingh K, Koo M, Vasanji A, et al (2007) Hyperproliferative apoptosis-resistant endothelial cells in idiopathic pulmonary arterial hypertension. Am J Physiol Lung Cell Mol Physiol 293(3): L548–54. 17526595 10.1152/ajplung.00428.2006

[5] Hubbi ME, Gilkes DM, Hu H, Kshitiz, Ahmed I, Semenza GL. (2014) Cyclin-dependent kinases regulate lysosomal degradation of hypoxia-inducible factor 1α to promote cell-cycle progression. Proc Natl Acad Sci USA 111(32): E3325–34. 10.1073/pnas.1412840111 25071185 PMC4136593

[6] Zeng XC, Liu FQ, Yan R, Yi HM, Zhang T, Wang GY, et al (2014) Downregulation of miR-610 promotes proliferation and tumorigenicity and activates Wnt/β-catenin signaling in human hepatocellular carcinoma. Mol Cancer 13: 261 10.1186/1476-4598-13-261 25491321 PMC4295306

[7] Shi X, Guo LW, Seedial SM, Si Y, Wang B, Takayama T, et al (2014) TGF-β/Smad3 inhibit vascular smooth muscle cell apoptosis through an autocrine signaling mechanism involving VEGF-A. Cell Death Dis 5: e1317 10.1038/cddis.2014.282 25010983 PMC4123076

[8] Karelina K, Liu Y, Alzate-Correa D, Wheaton KL, Hoyt KR, Arthur JS, et al (2015) Mitogen and stress-activated kinases 1/2 regulate ischemia-induced hippocampal progenitor cellproliferation and neurogenesis. Neuroscience 285: 292–302. 10.1016/j.neuroscience.2014.10.053 25451279 PMC5048677

[9] Sun LQ, Cairns MJ, Gerlach WL, Witherington C, Wang L, King A, et al (1999) Suppression of smooth muscle cell proliferation by a c-myc RNA-cleaving deoxyribozyme. J Biol Chem 274(24): 17236–41. 10358082 10.1074/jbc.274.24.17236

[10] Goncharova EA, Goncharov DA, Li H, Pimtong W, Lu S, Khavin I, et al (2011) mTORC2 is required for proliferation and survival of TSC2-Null Cells. Mol Cell Biol 31(12): 2484–2498. 10.1128/MCB.01061-10 21482669 PMC3133430

[11] Goncharov DA, Kudryashova TV, Ziai H, Ihida-Stansbury K, DeLisser H, Krymskaya VP, et al (2014) Mammalian target of rapamycin complex2(mTORC2) coordinates pulmonary artery smooth muscle cell metabolism, proliferation, and survival in pulmonary arterial hypertension. Circulation 129(8): 864–74. 10.1161/CIRCULATIONAHA.113.004581 24270265 PMC3968690

[12] Paddenberg R, Stieger P, von Lilien AL, Faulhammer P, Goldenberg A, Tillmanns HH, et al (2007) Rapamycin attenuates hypoxia-induced pulmonary vascular remodeling and right ventricular hyperplasia in mice. Respir Res 8: 15 17319968 10.1186/1465-9921-8-15 PMC1821322

[13] Goncharova EA. (2013) mTOR and vascular remodeling in lung diseases: current challenges and therapeutic prospects. FASEB J 27(5): 1796–807. 10.1096/fj.12-222224 23355268 PMC3633815

[14] Krymskaya VP, Snow J, Cesarone G, Khavin I, Goncharov DA, Lim PN, et al (2011) mTOR is required for pulmonary arterial vascular smooth cell proliferation under chronic hypoxia. FASEB J 25(6): 1922–33. 10.1096/fj.10-175018 21368105 PMC3101038

[15] Zhang P, Shan T, Liang X, Deng C, Kuang S. (2014) Mammalian target of rapamycin is essential for cardiomyocyte survival and heart development in mice. Biochem Biophys Res Commun 452(1): 53–9. 10.1016/j.bbrc.2014.08.046 25139234 PMC4382310

[16] Liu T, Yacoub R, Taliaferro-Smith LD, Sun SY, Graham TR, Dolan R, et al (2011) Combinatorial effects of lapatinib and rapamycin in triple-negative breast cancer cells. Mol Cancer Ther 10(8): 1460–9. 10.1158/1535-7163.MCT-10-0925 21690228 PMC4908959

[17] Eckerdt F, Beauchamp E, Bell J, Iqbal A, Su B, Fukunaga R, et al (2014) Regulatory effects of a Mnk2-eIF4E feedback loop during mTORC1 targeting of human medulloblastoma cells. Oncotarget 5(18):8442–51. 25193863 10.18632/oncotarget.2319 PMC4226695

[18] Liu Y, László C, Liu Y, Liu W, Chen X, Evans SC, et al (2010) Regulation of G(1) arrest and apoptosis in hypoxia by PERK and GCN2-mediated eIF2alpha phosphorylation. Neoplasia 12(1): 61–8. 20072654 10.1593/neo.91354 PMC2805884

[19] Liu X, Bennett RL, Cheng X, Byrne M, Reinhard MK, May WS Jr. (2013) PKR regulates proliferation, differentiation, and survival of murine hematopoietic stem/progenitor cells. Blood 121(17): 3364–74. 10.1182/blood-2012-09-456400 23403623 PMC3637012

[20] Bennett RL, Pan Y, Christian J, Hui T, May WS Jr. (2012) The RAX/PACT-PKR stress response pathway promotes p53 sumoylation and activation, leading to G_1_ arrest. Cell Cycle 11(2): 407–17. 10.4161/cc.11.2.18999 22214662 PMC3293386

[21] Bentley JK, Hershenson MB. (2008) Airway smooth muscle growth in asthma: proliferation, hyperplasia, and migration. Proc Am Thorac Soc 5(1): 89–96. 18094090 10.1513/pats.200705-063VS PMC2645305

[22] Anand P, Gruppuso PA. (2006) Rapamycin inhibits liver growth during refeeding in rats via control of ribosomal protein translation but not cap-dependent translation initiation. J Nutr 136(1): 27–33. 16365054 10.1093/jn/136.1.27 PMC1386153

[23] Li XW, Hu CP, Wu WH, Zhang WF, Zou XZ, Li YJ. (2012) Inhibitory effect of calcitonin gene-related peptide on hypoxia-induced rat pulmonary artery smooth muscle cells proliferation: role of ERK1/2 and p27. Eur J Pharmacol 679(1–3): 117–26. 10.1016/j.ejphar.2012.01.015 22306243

[24] Li XH, Peng J, Tan N, Wu WH, Li TT, Shi RZ, et al (2010) Involvement of asymmetric dimethylarginine and Rho kinase in the vascular remodeling in monocrotaline-induced pulmonary hypertension. Vascul Pharmacol 53(5–6): 223–229. 10.1016/j.vph.2010.09.002 20840872

[25] Bernard S, Eilers M. (2006) Control of cell proliferation and growth by myc proteins. Results Probl Cell Differ 42: 329–42. 16903216 10.1007/400_004

[26] Liu B, Luo XJ, Yang ZB, Zhang JJ, Li TB, Zhang XJ, et al (2014) Inhibition of NOX/VPO1 Pathway and Inflammatory Reaction by Trimethoxystilbene in Prevention of Cardiovascular Remodeling in Hypoxia-induced Pulmonary Hypertensive Rats. J Cardiovasc Pharmacol 63(6): 567–76. 10.1097/FJC.0000000000000082 24492474

[27] Chan SY, Loscalzo J. (2008) Pathogenic mechanisms of pulmonary arterial hypertension. J Mol Cardiol 44(1): 14–30. 10.1016/j.yjmcc.2007.09.006 PMC2234575 17950310

[28] Dahal BK, Cornitescu T, Tretyn A, Pullamsetti SS, Kosanovic D, Dumitrascu R, et al (2010) Role of epidermal growth factor inhibition in experimental pulmonary hypertension. Am J Respir Crit Care Med 181(2): 158–67. 10.1164/rccm.200811-1682OC 19850946

[29] Yang J, Li X, Li Y, Southwood M, Ye L, Long L, et al (2013) Id proteins are critical downstream effectors of BMP signaling in human pulmonary arterial smooth muscle cells. Am J Physiol Lung Cell Mol Physiol 305(4): L312–21. 10.1152/ajplung.00054.2013 23771884 PMC3891012

[30] Evangelisti C, Ricci F, Tazzari P, Tabellini G, Battistelli M, Falcieri E, et al (2011) Targeted inhibition of mTORC1 and mTORC2 by active-site mTOR inhibitors has cytotoxic effects in T-cell acute lymphoblastic leukemia. Leukemia 25: 781–791. 10.1038/leu.2011.20 21331075

[31] Houssaini A, Abid S, Mouraret N, Wan F, Rideau D, Saker M, et al (2013) Rapamycin reverses pulmonary artery smooth muscle cell proliferation in pulmonary hypertention. Am J Respir Cell Mol Biol 48(5): 568–77. 10.1165/rcmb.2012-0429OC 23470622 PMC4901163

[32] Wang W, Liu J, Ma A, Miao R, Jin Y, Zhang H, et al (2014) mTORC1 Is Involved in Hypoxia-Induced Pulmonary Hypertension through the Activation of Notch3. J Cell Physiol 229(12): 2117–25. 10.1002/jcp.24670 24825564

[33] Ogawa A, Firth AL, Yao W, Madani MM, Kerr KM, Auger WR, et al (2009) Inhibition of mTOR attenuates store-operated Ca2+ entry in cells from endarterectomized tissues of patients with chronic thromboembolic pulmonary hypertension. Am J Physiol Lung Cell Mol Physiol 297(4): L666–76. 10.1152/ajplung.90548.2008 19633069 PMC2770792

[34] Ogawa A, Firth AL, Ariyasu S, Yamadori I, Matsubara H, Song S, et al (2013) Thrombin-mediated activation of Akt signaling contributes to pulmonary vascular remodeling in pulmonary hypertension. Physiol Rep 1(7): e00190 10.1002/phy2.190 24744867 PMC3970741

[35] Humar R, Kiefer FN, Berns H, Resink TJ, Battegay EJ. (2002) Hypoxia enhances vascular cell proliferation and angiogenesis in vitro via rapamycin (mTOR)- dependent signaling. FASEB J 16(8): 771–80. 12039858 10.1096/fj.01-0658com

[36] Nührenberg TG, Voisard R, Fahlisch F, Rudelius M, Braun J, Gschwend J, et al (2005) Rapamycin attenuates vascular wall inflammation and progenitor cell promoters after angioplasty. FASEB J 19(2): 246–8. 15546959 10.1096/fj.04-2431fje

[37] Tang Z, Wang Y, Fan Y, Zhu Y, Chien S, Wang N. (2008) Suppression of c-Cbl tyrosine phosphorylation inhibits neointimal formation in balloon-injured rat arteries. Circulation 118(7): 764–72. 10.1161/CIRCULATIONAHA.107.761932 18663086

[38] Moss SC, Lightell DJ Jr, Marx SO, Marks AR, Woods TC. (2010) Rapamycin regulates endothelial cell migration through regulation of the cyclin-dependent kinase inhibitor p27Kip1. J Biol Chem 285(16): 11991–7. 10.1074/jbc.M109.066621 20097763 PMC2852937

[39] Sarbassov DD, Ali SM, Sengupta S, Sheen JH, Hsu PP, Bagley AF, et al (2006) Prolonged rapamycin treatment inhibits mTORC2 assembly and Akt/PKB. Mol Cell 22: 159–168. 16603397 10.1016/j.molcel.2006.03.029

[40] Harris TE, Chi A, Shabanowitz J, Hunt DF, Rhoads RE, Lawrence JC Jr. (2006) mTOR-dependent stimulation of the association of eIF4G and eIF3 by insulin. EMBO J 25(8):1659–68. 16541103 10.1038/sj.emboj.7601047 PMC1440840

[41] Sun LQ, Cairns MJ, Gerlach WL, Witherington C, Wang L, King A, et al (1999) Suppression of smooth muscle cell proliferation by a c-myc RNA-cleaving deoxyribozyme. J Biol Chem 274(24): 17236–41. 10358082 10.1074/jbc.274.24.17236

