# Supplementary figures and images for: A Critical Role of the mTOR/eIF2α Pathway in Hypoxia-Induced Pulmonary Hypertension
Source: PLoS One. 2015 Jun 29;10(6):e0130806. doi: 10.1371/journal.pone.0130806 (PMC4487252; doi:10.1371/journal.pone.0130806)

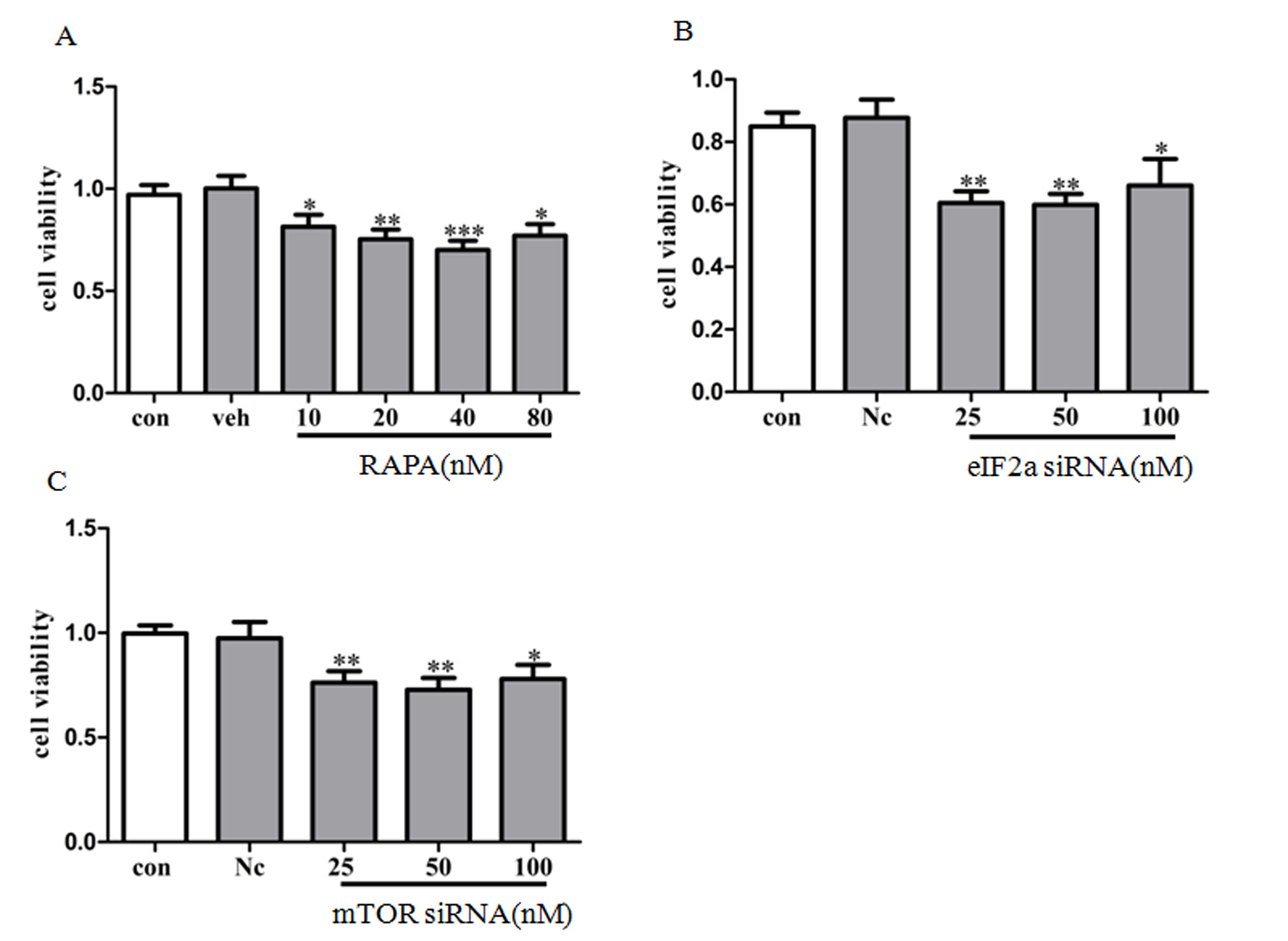

Supplement: S1 Fig — Cellular proliferation stimulated by 20% FBS (48 hours) was assayed in PASMCs treated with siRNA against mTOR or eIF2α, or in the presence of rapamycin. As shown in MTS experiments, cell proliferation was inhibited by either rapamycin (10, 20, 40, 80 nmol/L) (A), eIF2α siRNA (25, 50, 100 nmol/L) (B), or mTOR siRNA (25, 50, 100 nmol/L) (C). These results are in agreement with cell proliferation data acquired with hypoxia. Data represent the means ±S.E.M. n = 3. *P<0.05 vs. con; **P<0.01 vs. con; ***P<0.001 vs. con; # P<0.05 vs. hypoxia; ## P<0.01 vs. hypoxia. The above experiments were repeated three times with similar results. (TIF) [file pone.0130806.s001.tif]

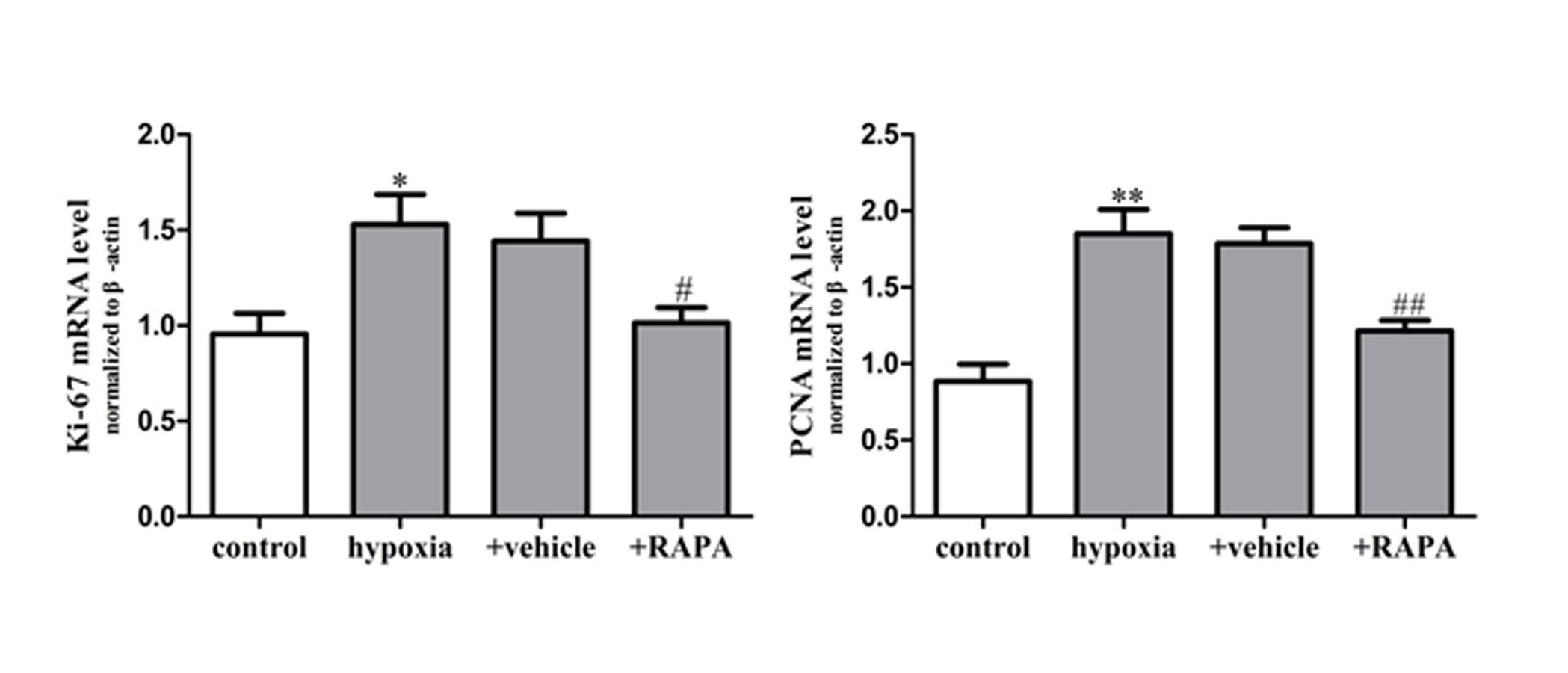

Supplement: S2 Fig — (A) The expression of Ki-67 mRNA was determined by real-time PCR. (B) The expression of PCNA mRNA was determined by real-time PCR. Data represent the means ±S.E.M. n = 3. *P<0.05 vs. control; **P<0.01 vs. control; # P<0.05 vs. hypoxia; ## P<0.01 vs. hypoxia. The above experiments were repeated three times with similar results. (TIF) [file pone.0130806.s002.tif]

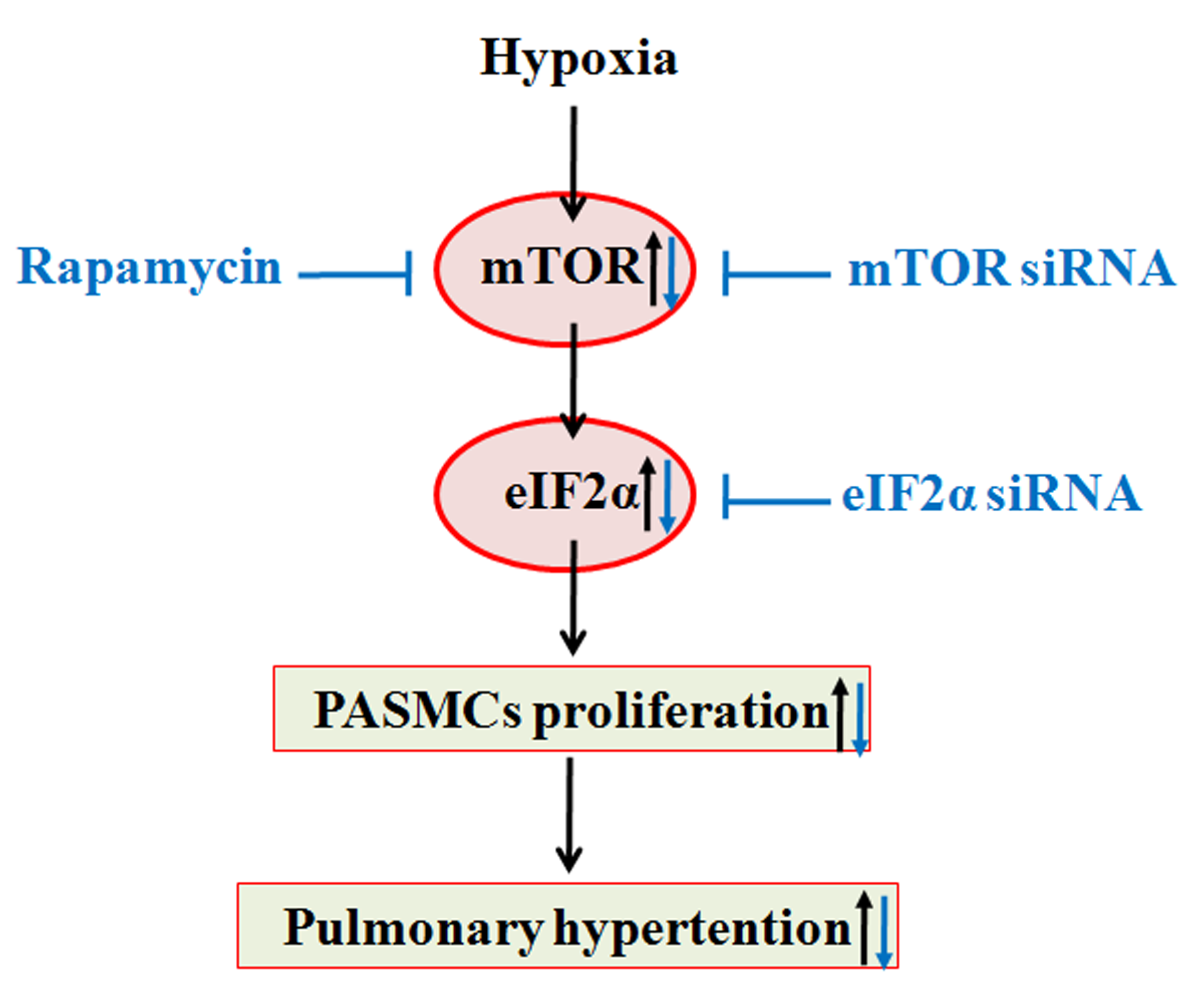

Supplement: S3 Fig — (TIF) [file pone.0130806.s003.tif]
